# Workshop with medical students on physicians’ earning opportunities, workload and job satisfaction increases the attractiveness of working self-employed and working in general practice

**DOI:** 10.1186/s12909-022-03191-3

**Published:** 2022-03-01

**Authors:** Alexander Heine, Anne-Kathrin Geier, Stefan Lippmann, Markus Bleckwenn, Thomas Frese, Tobias Deutsch

**Affiliations:** 1 Department of General Practice, Faculty of Medicine, University of Leipzig, Philipp-Rosenthal-Str. 55, 04103 Leipzig, Germany; 2 Institute of General Practice and Family Medicine, Martin-Luther-University Halle-Wittenberg, Halle/Saale, Germany

**Keywords:** Career choice, Medical students, Undergraduate medical education, Earning opportunities, Workload, Job satisfaction

## Abstract

**Background:**

Among the various factors identified as relevant for primary care career choice, financial considerations have been consistently shown to have an impact. In Germany, reliable and easily understandable information on physicians’ earning opportunities in self-employed settings is difficult to obtain for medical students, leading to substantial misperceptions that may negatively affect respective career considerations. This study investigated medical students’ evaluation of a 45-min evidence-based workshop on earning opportunities, workload and job satisfaction in different specialties and settings to examine its effect on the perceived attractiveness of working self-employed and working in general practice.

**Methods:**

The workshop was implemented as part of a mandatory general practice clerkship in the fourth study year (of six). Post-hoc evaluations of all participants between October 2017 and September 2018 (one cohort) were analysed cross-sectionally including descriptive statistics, subgroup comparisons and qualitative analysis of free-text answers regarding students’ main insights.

**Results:**

Response rate was 98.1% (307/313). Participants were on average 25.0 years old, and 68.3% were women. Based on a ten-point scale ranging from 1 = ’no influence’ to 10 = ’very big influence’, 91.9% confirmed at least some (> = 2) and 57.3% a rather high (> = 5) influence of earning expectations on their career choice process. Regarding the workshop, 86.1% were overall satisfied, and 89.5% indicated they had gained new insights, primarily regarding earning opportunities in different specialties and work settings, and frequently regarding job satisfaction, workload and the structure of revenues and expenditures in a doctor’s office (according to qualitative analysis). In the opinion of 89.8% of students, the provided learning content should be part of the undergraduate curriculum. More than half of participants reported an increase regarding the attractiveness of working self-employed and working as a general practitioner, most frequently regarding earning opportunities, but also in general and in respect to job satisfaction, cost–benefit ratio and workload. This increase was significantly higher among students favouring or at least considering a general practice career.

**Conclusions:**

The workshop and its content were appreciated by the students and showed clear potential to usefully complement undergraduate curricula aiming at increasing or reinforcing students’ interest in working self-employed and working in general practice.

**Supplementary Information:**

The online version contains supplementary material available at 10.1186/s12909-022-03191-3.

## Background

Like many other countries, Germany is currently facing a growing shortage of primary care physicians, particularly in general practice [1–5]. To prevent a future undersupply, more medical students need to be convinced to enter general practice careers. To reach this goal, collective efforts must be undertaken by political decision-makers, stakeholders from the health care system and medical faculties, addressing various influencing factors at different levels and at different stages of medical education and physicians’ professional growth [6–8].

Previous research has described medical students’ specialty choice as a complex process influenced by demographical, educational and environmental factors; personal interests and preferences; experiences; and lifestyle considerations [6, 7, 9, 10]. Among the many factors identified as relevant for primary care career choice, financial considerations have also been consistently shown to have a substantial impact [6, 10–13]. Accordingly, we found in a prior study of our own research group that the expectation of inadequate earning opportunities, in general or in relation to workload, was among the most frequently mentioned motives of German medical graduates to reject a previously considered career as a general practitioner (GP) [14]. However, we demonstrated in another study that although German medical students regard financial aspects as clearly important for their career choice, they are often insufficiently informed about physicians’ earnings (particularly in outpatient care) and substantially underestimate earning opportunities in self-employed settings and in general practice [13]. This can be explained to a large extent by a lack of easily accessible, reliable, easily understandable and comparable statistics regarding the attainable earnings per physician working in self-employed settings in Germany [15]. Most German GPs work self-employed in their own outpatient (mostly single) practices, and instead of receiving a salary (like in a hospital), they are remunerated mainly based on a fee-for-service system financed by patients’ statutory or private health insurances. However, although self-employed GPs’ workload is high, both working hours and net income do not substantially differ from other similar specialists [16]. In summary, there is some risk that German medical graduates reject careers in general practice partly due to misperceptions regarding earning opportunities. Consequently, the provision of sound information for medical students and residents regarding finances in self-employed settings and general practice has been suggested by some authors [11, 13, 17–19].

Based on this knowledge, we designed a workshop that provided evidence-based information on revenues and expenditures in German outpatient practices and earning opportunities in different specialties and settings (outpatient vs. hospital, employed vs. self-employed, big city vs. rural area), as well as addressed issues such as workload and job satisfaction in different contexts. The 45-min interactive workshop was integrated as final event into a mandatory two-week general practice clerkship in the fourth study year (of six). The workshop aimed to enhance transparency regarding physicians’ earnings and help students make a well-informed career choice in respect to financial considerations (for concrete teaching goals, see Methods section).

In this study, we wanted to find out how the workshop was evaluated by the students, if students gained new insights from the workshop and if students rated the provided information as relevant for their future job activities. Furthermore, we wanted to investigate whether the workshop could enhance the attractiveness of working self-employed as well as the attractiveness of working as a general practitioner in general, and in terms of earning opportunities, workload and job satisfaction.

## Methods

### Sampling and design

The present cross-sectional data are based on fourth-year (of six) medical students’ post-hoc evaluations of a workshop on physicians’ earning opportunities, workload and job satisfaction. The workshop was conducted with small groups of 8 to 15 students at the end of their mandatory two-week general practice clerkship at the University of Leipzig, Germany. All students who completed the clerkship between October 2017 and September 2018 (one cohort) took part in the workshop and were subsequently asked to answer an anonymous paper-based questionnaire on a voluntary basis.

### Content of the workshop (curricular intervention)

The workshop had a duration of 45 to 60 min (maximum) and followed an interactive approach (e.g., working with presentation cards, small group discussions, estimation questions). Throughout the study period, the workshop was consistently led by the same teacher (AH), who was a general practice resident at this time. The workshop’s teaching goals were the following:

**Teaching Goals: After participation in the workshop, medical students know:**

- sources of income of (German) physicians self-employed in their own outpatient practice, and differences between specialities regarding their contribution to the overall income
- fundamentals of the German fee-for-service system regarding patients with statutory or private health insurances, fee schedule for physicians, and individual health services
- fundamentals of budgeting in (German) outpatient care
- the structure of revenues and costs in a doctor’s practice considering different specialties and fixed and variable costs
- the difference between gross and net income
- the monthly net income of different specialties in self-employed outpatient care (median and quartiles)
- differences regarding self-employed earning opportunities in big cities versus rural areas
- differences regarding earning opportunities when working employed in a hospital versus self-employed in own practice
- the amount and structure of weekly working time for different specialties in self-employed outpatient care (time overall, time working with patient, time for bureaucracy, further education, practice management, etc.) in comparison to working employed in a hospital
- study results regarding the job satisfaction of physicians working in different settings and specialties

All comparable figures used in the workshop were derived from officially available sources regarding German physicians’ earnings and workload in different settings, as well as studies on job satisfaction of physicians (e.g., [16, 20]). The most important source was the ZI (Central Research Institute of Ambulatory Health Care in Germany) practice panel. This panel annually analyses the economic situation, workload and job satisfaction of self-employed physicians of different specialties for official purposes based on data from thousands of practices [16]. By using data of the participating physicians’ tax consultants, the ZI practice panel can be seen as the best and most comprehensive source regarding German self-employed physicians’ income.

### Questionnaire

The questionnaire used in this study was self-developed by a multidisciplinary team consisting of a general practitioner, a general practice resident, a psychologist and an economist. It contained items addressing relevant sociodemographic variables as well as career considerations, the estimated influence of earning expectations on the own career choice process and search for information on future earnings previous to the workshop. Furthermore, students were asked to assess the workshop’s structure and benefit, as well as its impact on the attractiveness of working self-employed (own practice) in general and particularly in general practice in terms of workload, job satisfaction and earning opportunities. An additional open-ended question (free-text answer) enquired the main insights from the workshop from the students’ perspective. To enhance face validity, comprehensibility and usability, the pre-final version of the questionnaire was pre-tested with three medical students in advanced study years (target group) following the method of concurrent think aloud (CTA). The results of the pre-testing procedure led to minor adjustments regarding form and wording. An English translation of the final questionnaire version is provided in Additional file 1.

### Statistical analysis

Data was analysed using IBM SPSS Statistics 24 for Windows. Frequencies were presented as %_valid_ (n_absolute_/n_valid_) considering missing values for single items. Continuous variables were presented as mean ± standard deviation (SD) complemented by median and quartiles, if useful. In addition to descriptive statistics, Mann–Whitney U-Test was used to analyse differences in central tendency between independent groups. Frequency distributions were compared using Chi-square test and Fisher’s exact test as appropriate. Statistical significance was assumed for *p* < 0.05.

### Qualitative analysis of free-text answers

The participants’ free-text answers regarding their main insights from the workshop were analysed following the content analysis approach by Mayring [21]. In a first step, a category system was developed independently by two scientists (a physician and a psychologist) following an inductive approach and including the whole material. Subsequently, consensus was found for all differences, and the material was reassigned to the final categories. Applicable categories were used only once per person. To assess the reliability of the findings, a third and previously uninvolved rater assigned the raw data once again. Agreement was 88.6%, which can be considered as very good. Finally, absolute and relative frequencies of mentioning the single categories were calculated.

## Results

Of the 313 students who completed the mandatory two-week general practice clerkship during the study period, 307 returned a completed questionnaire (response rate = 98.1%). The mean age of the participants was 25.0 ± 3.7 years, and 68.3% (209/306) were female. Detailed sample characteristics are displayed in Table 1. Living in a big city in the future was imaginable for 63.2% (194/307) of the students, 65.8% (202/307) could imagine living in a small-town and 37.5% (115/307) in a rural area. General practice was the currently favoured career option for 14.0% (42/300) and at least a considered option for 38.7% (113/292) of participants.

**Table 1:** Sample characteristics

Variable	valid (*n*)*	*n* (%)**
Age [mean ± SD]	305	25.0 ± 3.7
Female gender	306	209 (68.3)
In a relationship	302	149 (49.3)
Has children	297	20 (6.7)
At least one parent with higher education degree	299	235 (78.6)
Being a physician’s child	306	74 (24.2)
Family or friends in general practice	307	85 (27.7)
Family or friends working as an office-based physician	306	118 (38.6)
Mainly grown up in …	306	
… big city		108 (35.3)
… small town		100 (32.7)
… rural area		98 (32.0)
Previously completed education in a medical occupation	304	75 (24.7)
Has already worked in social/ medical field	297	110 (37.0)

^*^ *n* varies due to missing values

^**^ Unless otherwise indicated

**Fig. 1:**
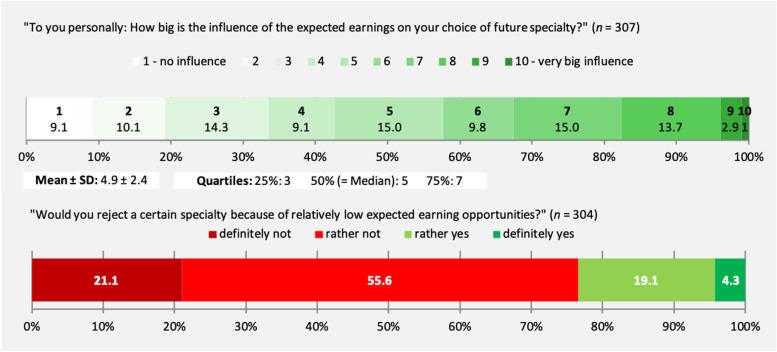
Influence of expected earning opportunities on career choice as perceived by study participants

Students’ ratings regarding the self-perceived influence of expected earning opportunities on their personal career choice are presented in Fig. 1. While nine out of ten students reported some kind of influence (> = 2 on the used ten-point scale), more than half of participants rated this influence as rather high (> = 5 to 10). Nearly a quarter of students would reject a certain specialty due to relatively low expected earnings. Altogether, 45.6% (140/307) indicated they had already gained information on future earning opportunities prior to the course. Of these 140 participants with previous information, 130 indicated the sources they have used. In total, they made 168 single statements, which could be categorised as follows (descending order by frequency): internet, not specified, 51.5% (67/130); personally known doctors/doctors during clerkships, 30.0% (39/130); internet, tariff agreements, 29.2% (38/130); lectures/courses at university, 5.4% (7/130); internet, available official statistics, 4.6% (6/130); internet, salary comparisons/rankings, 3.1% (4/130); medical associations, 2.3% (3/130); commercial investment counselling, 1.5% (2/130); conferences, 0.8% (1/130); online journals, 0.8% (1/130).

Altogether, 22.5% (69/306) of students had discussed the earnings of self-employed GPs with their GP teacher during the two-week clerkship before the workshop. Of these 69 students, 16 reported that concrete figures were provided, corresponding to an overall proportion of 5.2% (16/306) of the entire sample.

**Fig. 2:**
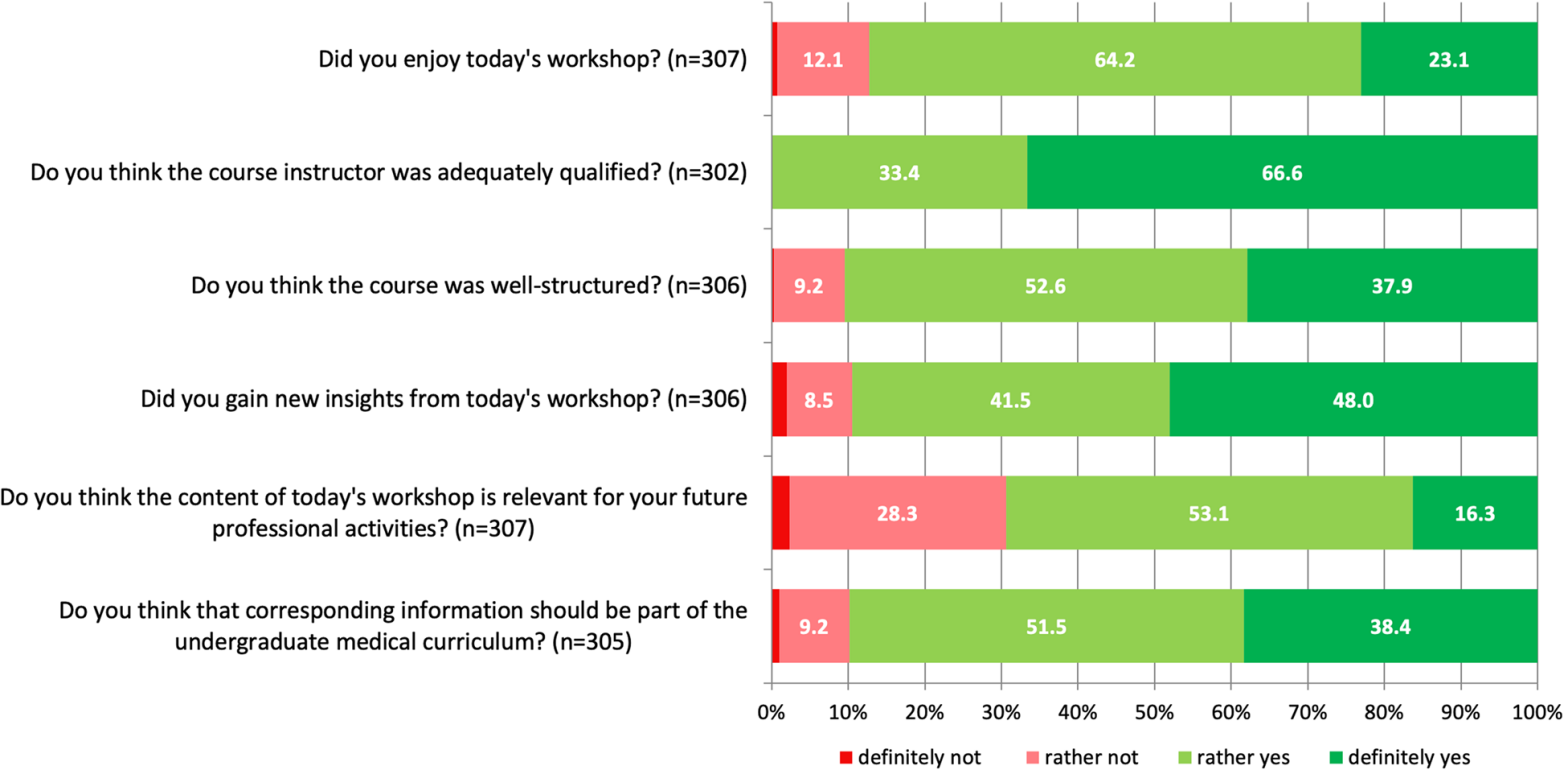
General evaluation of the workshop and perceived benefit

Regarding overall satisfaction with the course, 25.5% (75/294) of participants stated to be ‘very satisfied’; 60.5% (178/294) ‘rather satisfied’; 9.2% (27/294) ‘rather unsatisfied’; and 4.8% (14/294) ‘very unsatisfied’. Further results addressing the students’ overall evaluation of the course as well as their perceived benefit are shown in Fig. 2. There were no statistically significant frequency differences regarding the proportion of participants reporting to have gained new insights from the course (‘definitely or rather yes’ vs. ‘rather or definitely not’) depending on the variables sex, being a physician’s child, having family or friends in general practice, having family or friends working as a self-employed physician and favouring or at least considering a career in general practice (Chi^2^ = 0.008–3.382; *p* = 0.066–0.928). We also found no significant frequency differences regarding the proportion of students stating that the provided information should be part of the medical curriculum (‘definitely or rather yes’ vs. ‘rather or definitely not’) depending on the same variables (Chi^2^ = 0.292–0.585; *p* = 0.444–0.778). The workshop’s group size was ‘just right’ for 97.4% (297/305), ‘too small’ for 2.0% (6/305) and ‘too big’ for 0.7% (2/305). The length of the workshop was ‘just right’ for 93.5% (286/306), ‘too long’ for 5.2% (16/306) and ‘too short’ for 1.3% (4/306) of the participants.

**Fig. 3:**
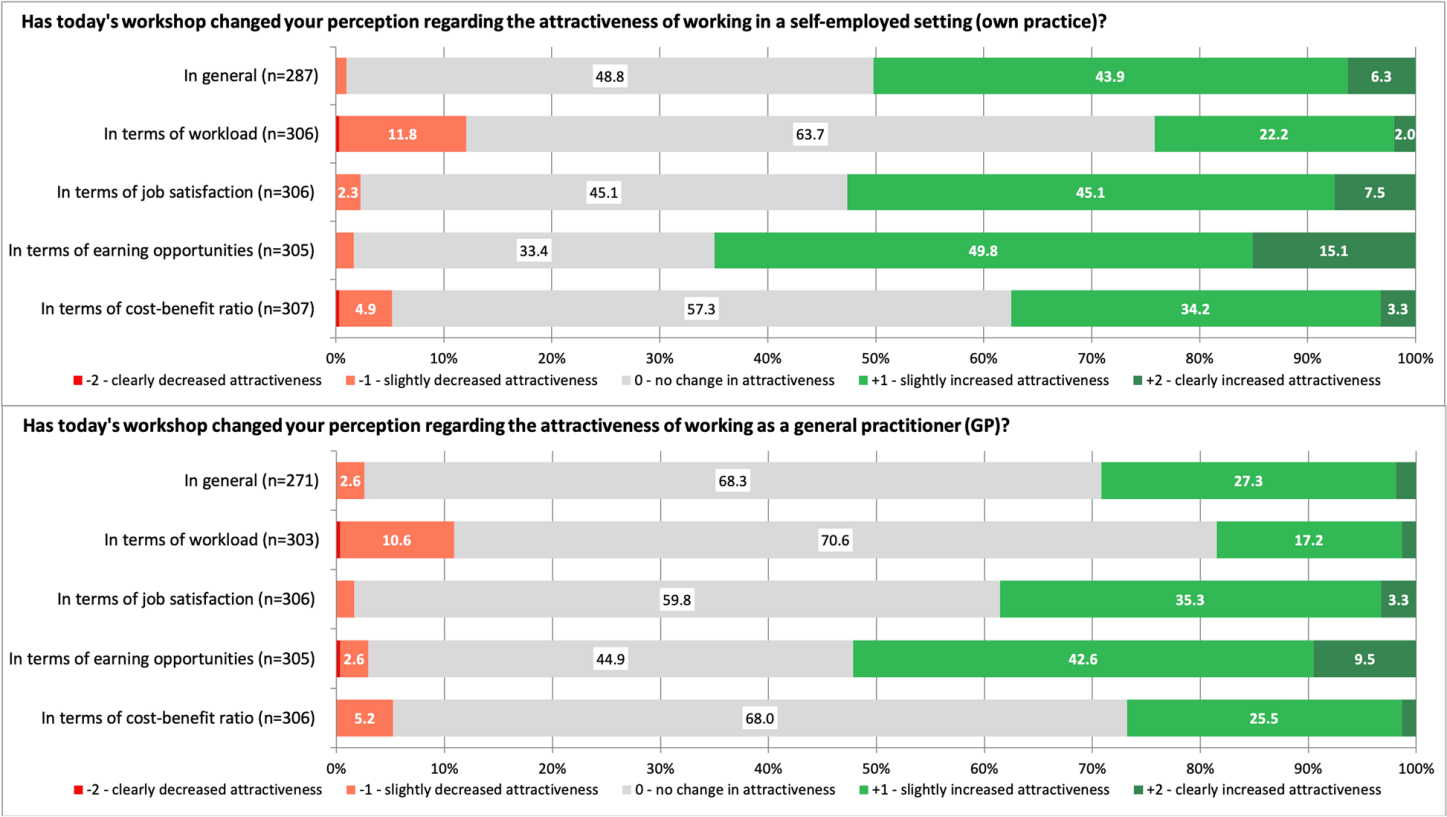
Changes in medical students’ perceptions toward self-employment and working as a general practitioner following the workshop

Changes in the students’ perceptions regarding the attractiveness of working in a self-employed setting and working as a general practitioner because of the course are displayed in Fig. 3. Compared to their female counterparts, male participants reported a slightly but significantly higher increase in the perceived attractiveness of working in a self-employed setting and in general practice in terms of the cost–benefit ratio (mean ± SD: 0.5 ± 0.7 vs. 0.3 ± 0.6, *p* = 0.008; 0.3 ± 0.6 vs. 0.2 ± 0.5, *p* = 0.007, scale from -2 ‘clearly decreased’ to + 2 ‘clearly increased’). We found no sex-related differences regarding a change in perceived attractiveness in general and in terms of workload, job satisfaction, and earning opportunities. Furthermore, there were no significant differences regarding any change in attractiveness depending on whether being a physician’s child or having family or friends working as a self-employed physician. Students having family or friends in general practice reported a slightly but significantly higher increase in the perceived attractiveness of working in a self-employed setting in terms of workload (mean ± SD: 0.3 ± 0.7 vs. 0.1 ± 0.6, *p* = 0.017) and job satisfaction (mean ± SD: 0.7 ± 0.7 vs. 0.5 ± 0.6, *p* = 0.008), as well as in the perceived attractiveness of working in general practice in terms of job satisfaction (mean ± SD: 0.5 ± 0.7 vs. 0.4 ± 0.5, *p* = 0.021). Differences between students who favoured or at least considered general practice as a future career and those who did not regarding changes in attractiveness of working self-employed or as a general practitioner are shown in Table 2.

**Table 2:** Comparison between students who favoured or considered a GP career and those who did not

Has today’s workshop changed your perception regarding the attractiveness of …	**GP career is favoured or considered**		**Other students**		*p*
	*n*	mean ± SD	*n*	mean ± SD	
**working in a self-employed setting? **^a^					
… in general	107	0.7 ± 0.7	167	0.4 ± 0.6	< 0.001
… in terms of workload	113	0.1 ± 0.6	178	0.2 ± 0.6	0.304
… in terms of job satisfaction	112	0.8 ± 0.7	179	0.5 ± 0.6	< 0.001
… in terms of earning opportunities	112	0.9 ± 0.7	178	0.7 ± 0.7	0.004
… in terms of the cost–benefit ratio	113	0.5 ± 0.6	179	0.3 ± 0.7	0.047
**… working as a general practitioner? **^a^					
… in general	99	0.6 ± 0.6	159	0.1 ± 0.4	< 0.001
… in terms of workload	111	0.1 ± 0.7	177	0.1 ± 0.5	0.468
… in terms of job satisfaction	113	0.6 ± 0.6	178	0.3 ± 0.5	< 0.001
… in terms of earning opportunities	112	0.8 ± 0.7	178	0.4 ± 0.7	< 0.001
… in terms of the cost–benefit ratio	113	0.4 ± 0.6	178	0.1 ± 0.5	< 0.001

^a^** Scale:** -2 = clearly decreased attractiveness; -1 = slightly decreased attractiveness; 0 = no change in attractiveness; + 1 = slightly increased attractiveness; + 2 = clearly increased attractiveness

A categorisation of the participants free-text answers (qualitative content analysis) on the question ‘What were the most important insights for you from today’s course?’ is presented in Table 3. Based on a subsequent frequency analysis of students with corresponding statements, the resulting categories are displayed in a descending order by frequency of being mentioned.

**Table 3:** Medical students’ main insights from the workshop (qualitative analysis complemented by subsequent frequency analysis)

**What were the most important insights for you from today’s course?** (*n* = 253) (descending order by frequencies of persons with corresponding statements)	
**Response categories** Insights regarding …	**Frequencies**
Earning opportunities of different specialties in self-employed settings – in general and compared to hospital	56.1% (142/253)
Job satisfaction in different specialties and settings	27.7% (70/253)
Working hours and bureaucracy in different specialties and settings	19.0% (48/253)
Payment system and structure of revenues and expenditures in self-employed settings	15.4% (39/253)
Perception that general practitioners earn good money or earn more money than previously expected	13.8% (35/253)
Pros and cons comparison of self-employed setting vs. working in hospital	6.7% (17/253)
Particularities of working self-employed in rural areas (e.g., specific financial support, earnings)	5.1% (13/253)
Confirmation of the importance of facing these career considerations	2.4% (6/253)
Organisational framework conditions of working self-employed	1.6% (4/253)
Confirmation of pre-existing career considerations regarding specialty or setting	1.6% (4/253)
Personal insight that money is not decisive for career choice	1.2% (3/253)
Discovery of new sources of information	1.2% (3/253)
Increased attractiveness of working self-employed	0.8% (2/253)
Confirmation of pre-existing knowledge	0.8% (2/253)
Other (e.g., vague comments that could not be specified any further or individual opinions not answering the question)	5.9% (15/253)

## Discussion

### Summary of the main findings

The workshop was rated highly by students. A vast majority were overall satisfied, welcomed the learning content and stated to have gained relevant new insights, mainly regarding earning opportunities in different specialties and work settings, and frequently regarding job satisfaction, workload, and the structure of revenues and expenditures in private practice. A substantial proportion of students reported that participation in the workshop positively influenced their perception regarding the attractiveness of working self-employed in general as well as working as a GP. This effect was significantly higher among those students favouring or at least considering general practice as a future career option.

### Interpretation and comparison with the literature

Before discussing the new insights gained by this study, it is important to note that the students in our sample confirmed the important influence of financial considerations on their process of specialty choice, with more than half of them rating this influence as rather high. These results reproduce nearly exactly the findings of our previous work, which was the starting point for the development of the workshop investigated in this study [13]. Moreover, these results are in accordance with the international literature on career decision-making in medicine, which has consistently reported an existing influence of income expectations [6, 7, 10, 22–24]. Previous German studies have also underlined the relevance of perceived financial opportunities for the choice of a specialty or the decision to work self-employed by establishing a personal practice [14, 17, 25]. We could also confirm our previous findings that despite the highly rated impact of financial considerations, only a small number of fourth year medical students had already gained concrete information on future earning opportunities by using sources of information that are frequently not representative [13]. Another new aspect added by the present study is that during GP clerkships, students and GP teachers rarely talk about earning opportunities and even less about concrete figures.

Against this background, it seems hardly surprising that our workshop addressing physicians’ earning opportunities, workload and job satisfaction in different settings was highly welcomed by students. The workshop provided an opportunity for discussion and mutual exchange of knowledge and experience regarding a rarely discussed topic. This may have compensated for the underrepresentation of the subject during the clerkship. Furthermore, as there are hardly any opportunities for exchange with fellow students in individually completed GP clerkships, the interactive nature of the workshop was certainly of additional value for the students and may have contributed to the high level of overall satisfaction. Besides high overall satisfaction, nine out of ten students stated to have gained new insights and indicated this kind of learning content should be part of the undergraduate medical curriculum. Although we found no directly comparable literature regarding these findings, a previous study by Kohlhaas et al. at least indicated a general open-mindedness of German medical undergraduates regarding teaching content addressing entrepreneurial and business management issues [26].

We found no associations between having gained new insights from our workshop or valuing its content depending on sex, current career considerations or having physicians or GPs among family or friends. These findings imply that a broad spectrum of students would benefit from respective teaching content, not only those with specific interests or those without personal access to first-hand information.

Considering the important influence of financial considerations on the process of career choice confirmed in this study, previously reported frequent misperceptions regarding earnings in self-employed settings among German medical students [13], and a poor availability of easily accessible, reliable, easily understandable and comparable statistics regarding the attainable earnings of self-employed physicians in Germany, we were interested in how the information provided in our workshop would affect the perceived attractiveness of working self-employed as well as working as a GP. Our results show that the attractiveness of these career options increased for a substantial amount of the participants, particularly regarding earning opportunities. Few students reported decreased attractiveness, mainly with regard to workload. However, as these are short-term effects directly after our workshop, future research should investigate the long-term effects of this kind of intervention, as well as its contribution toward positively influencing primary care career choices during undergraduate medical education.

Except for the issue of workload, we found a significant higher increase in the perceived attractiveness of working self-employed or as a GP among students who currently favoured or at least considered a future GP career. These findings imply that the provided teaching content is particularly suitable to reinforce GP career considerations among students with at least an existing interest in primary care.

With regard to content, students who participated in our workshop reported new insights mainly regarding earning opportunities in different specialties and work settings, but also frequently regarding job satisfaction, workload and revenues and expenditures in private practice. We interpret this as evidence of the need to complement information on earning opportunities with further information to help students see the bigger picture and evaluate the information in context.

### Strengths and limitations

This study addresses an important topic of current political relevance in many countries. It offers a new approach that may be integrated with further development of curricular initiatives aimed at increasing the numbers of graduates choosing primary care careers. The very high response rate and the plausible distribution of sample characteristics increase the representativeness of the sample. However, the fact that our study was conducted at only one medical school limits the generalisability of our findings. Moreover, with our study design, we could show only short-term effects of our intervention on the perceived attractiveness of working self-employed or working as a GP. Further research is needed to investigate to what extent it will actually contribute to impact career choice. Furthermore, in our study we asked the participants to evaluate perceived changes regarding the attractiveness of working in a self-employed setting and in general practice due to our workshop. However, it should be taken into account that the workshop was the final event of a two-week GP clerkship and despite asking for the workshop’s effect the clerkship itself might have influenced the overall attitude towards general practice and students’ open-mindedness regarding the workshop content. This should be kept in mind when interpreting some of our results. As a further limitation, it should be considered that some premises in our study might be specific to the German context to a certain extent. Finally, it should be kept in mind that financial considerations are of course only one factor among many that influence career choice. However, targeted education may dispel common misperceptions to enhance transparency and help students consider financial issues on a well-informed and realistic basis.

## Conclusions

In addition to confirming the important influence of financial considerations on the process of medical students’ career choice, our results show that a 45-min workshop providing well-founded information on earning opportunities, workload and job satisfaction during undergraduate education is both appreciated by students and has the potential to increase the attractiveness of working self-employed and working as a GP. The workshop benefitted a broad range of students, not only for those with specific career interests or those without private access to first-hand information through family or friends working as self-employed physicians or GPs. Although the workshop showed a clear short-term effect on students’ perceptions, long-term effects on career choice after graduation still need to be investigated. The results are of interest for GPs involved in teaching undergraduates, as well as people planning undergraduate curricular activities aiming to affect medical students career considerations, particularly in general practice.

## Supplementary Information

**Additional file 1.** English translation of the analysed questionnaire items.

## Data Availability

The datasets used for the current study are available from the corresponding author on reasonable request.

## Abbreviations

CTA: Concurrent Think Aloud
GP: General Practitioner
SD: Standard Deviation
ZI: Zentralinstitut für die kassenärztliche Versorgung in Deutschland (Central Research Institute of Ambulatory Health Care in Germany)
