# Supplementary material for: Workshop with medical students on physicians’ earning opportunities, workload and job satisfaction increases the attractiveness of working self-employed and working in general practice
Source: BMC Med Educ. 2022 Mar 1;22:134. doi: 10.1186/s12909-022-03191-3 (PMC8887135; doi:10.1186/s12909-022-03191-3)
Supplement: Supplementary file 1 — Additional file 1. English translation of the analysed questionnaire items. [file 12909_2022_3191_MOESM1_ESM.docx]

**Additional File 1 – English translation of the analysed questionnaire items**

**Questionnaire referring to the workshop ‘Earning opportunities in self-employed settings’**

IMPORTANT: Please answer all questions completely. Thank you!

| **Personal information** | | | | | | | | | | | | | | | | | | | | | | | | | | | | | | | | | | | |
| --- | --- | --- | --- | --- | --- | --- | --- | --- | --- | --- | --- | --- | --- | --- | --- | --- | --- | --- | --- | --- | --- | --- | --- | --- | --- | --- | --- | --- | --- | --- | --- | --- | --- | --- | --- |
| Age | | | | \|__\|__\| years | | | | | | | | | Sex | | | | | | | | | ○ male | | | | ○ female | | | | | | | | | |
| Are you in a relationship? | | | | ○ yes | | | | | | ○ no | | | Do you have children? | | | | | | | | | ○ yes | | | | ○ no | | | | | | | | | |
| Do at least one of your parents have a higher education degree? | | | | | | | | | | | | | | | | | | | | | ○ yes, one or both | | | | | | | | ○ no, none | | | | | | |
| Is (at least) one of your parents a physician? | | | | | | | | | | | | | ○ yes | | | | | | | | | | ○ no | | | | | | | | | | | | |
| Do you have family or friends working in general practice? | | | | | | | | | | | | | ○ yes | | | | | | | | | | ○ no | | | | | | | | | | | | |
| Do you have family or friends working as an office-based physician? | | | | | | | | | | | | | | | | | | | | | | | ○ yes | | | | | | | | ○ no | | | | |
| Where did you mainly grow up? | | | | | | | | | | | | | ○ big city | | | | | ○ small town | | | | | | | ○ rural area | | | | | | | | | | |
| I have a qualification in a medical vocational education. | | | | | | | | | | | | | | | | ○ yes, degree in: ____________________ | | | | | | | | | | | | | | | | | | ○ no | |
| I have already worked in a social or medical field: | | | | | | | | | | | | | | | | | | | | | | | | | | | | | | | | | | | |
| ○ Federal volunteer service/Community service | | | | | | | | | | | | ○ other, _____________________ | | | | | | | | | | | | | | | | | | | | | | | ○ no |
| Where can you imagine living in the future (in the long run, multiple answers possible)? | | | | | | | | | | | | | | | | | | | | | | | | | | | | | | | | | | | |
| ○ big city | | | | | | ○ small town | | | | | | | | | | | | | | ○ rural area | | | | | | | | | | | | | | | |
| **Information on career considerations** | | | | | | | | | | | | | | | | | | | | | | | | | | | | | | | | | | | |
| My currently favoured medical specialty is: | | | | | | | | | | | | | | | | | | | | | | | | | | | | | | | | | | | |
| ○ | General practice | | | | | ○ | | other specialty: ______________________________ | | | | | | | | | | | | | | | | | | | | | | ○ | | do not know yet | | | |
| Many students have not finally decided on their medical specialty but consider several options. Please rank the specialties you consider as career options and begin with your favoured option (maximum of four): | | | | | | | | | | | | | | | | | | | | | | | | | | | | | | | | | | | |
| First choice: | | | | | | _______________________________________________ | | | | | | | | | | | | | | | | | | | | | | | | | | | | | |
| Second choice: | | | | | | _______________________________________________ | | | | | | | | | | | | | | | | | | | | | | | | | | | | | |
| Third choice: | | | | | | _______________________________________________ | | | | | | | | | | | | | | | | | | | | | | | | | | | | | |
| Fourth choice: | | | | | | _______________________________________________ | | | | | | | | | | | | | | | | | | | | | | | | | | | | | |
| To you personally: How big is the influence of the expected earnings on your choice of future specialty? | | | | | | | | | | | | | | | | | | | | | | | | | | | | | | | | | | | |
| no influence | | ➀ | ➁ | | | | ➂ | | ➃ | | ➄ | | | ➅ | | | ➆ | | ➇ | | | | | ➈ | | | ➉ | | | | | | very big influence | | |
| Would you reject a certain specialty because of relatively low expected earning opportunities? | | | | | | | | | | | | | | | | | | | | | | | | | | | | | | | | | | | |
| ○ definitely not | | | | ○ probably not | | | | | | | | | | ○ probably yes | | | | | | ○ definitely yes | | | | | | | | | | | | | | | |
| **Details on prior information** | | | | | | | | | | | | | | | | | | | | | | | | | | | | | | | | | | | |
| Had you already been looking for information on future earning opportunities (before this workshop)? | | | | | | | | | | | | | | | | | | | | | | | ○ yes | | | | | ○ no | | | | | | | |
| If yes, which sources of information did you use?  . _ . | | | | | | | | | | | | | | | | | | | | | | | | | | | | | | | | | | | |
| Did you talk with your general practice preceptor about earnings of self-employed general practitioners during your mandatory two-week general practice clerkship? | | | | | | | | | | | | | | | | | | | | | | | ○ yes | | | | | ○ no | | | | | | | |
| If yes, did you talk about concrete figures? | | | | | | | | | | | | | | | | | | | | | | | ○ yes | | | | | ○ no | | | | | | | |
| **Perceived benefit of today’s workshop** | | | | | | | | | | | | | | | | | | | | | | | | | | | | | | | | | | | |
| Did you enjoy today’s workshop? | | | | | | | | | | | | | | | | | | | | | | |  | | | | |  | | | | | | | |
| ○ definitely not | | | | | ○ rather not | | | | | | | | | | ○ rather yes | | | | | | | | ○ definitely yes | | | | | | | | | | | | |
| Do you think the course instructor was adequately qualified? | | | | | | | | | | | | | | | | | | | | | | |  | | | | |  | | | | | | | |
| ○ definitely not | | | | | ○ rather not | | | | | | | | | | ○ rather yes | | | | | | | | ○ definitely yes | | | | | | | | | | | | |
| Do you think the course was well-structured? | | | | | | | | | | | | | | | | | | | | | | |  | | | | |  | | | | | | | |
| ○ definitely not | | | | | ○ rather not | | | | | | | | | | ○ rather yes | | | | | | | | ○ definitely yes | | | | | | | | | | | | |
| Did you gain new insights from today’s workshop? | | | | | | | | | | | | | | | | | | | | | | |  | | | | |  | | | | | | | |
| ○ definitely not | | | | | ○ rather not | | | | | | | | | | ○ rather yes | | | | | | | | ○ definitely yes | | | | | | | | | | | | |
| Do you think the content of today’s workshop is relevant for your future professional activities? | | | | | | | | | | | | | | | | | | | | | | |  | | | | |  | | | | | | | |
| ○ definitely not | | | | | ○ rather not | | | | | | | | | | ○ rather yes | | | | | | | | ○ definitely yes | | | | | | | | | | | | |
| Do you think that corresponding information should be part of the undergraduate medical curriculum? | | | | | | | | | | | | | | | | | | | | | | |  | | | | |  | | | | | | | |
| ○ definitely not | | | | | ○ rather not | | | | | | | | | | ○ rather yes | | | | | | | | ○ definitely yes | | | | | | | | | | | | |

| Group size was: | o just right | o too big | o too small |
| --- | --- | --- | --- |
| Workshop length was: | o just right | o too long | o too short |

| **Has today’s workshop changed your perception regarding the attractiveness of working in a self-employed setting (own practice)?** | | | | | | | | | | |
| --- | --- | --- | --- | --- | --- | --- | --- | --- | --- | --- |
|  | | The attractiveness has… | | | | | | | | |
|  | | **-2**  clearly decreased | | **-1**  slightly decreased | | **0**  not changed | | **+1**  slightly increased | | **+2**  clearly increased |
| **A) In general** | | ○ | | ○ | | ○ | | ○ | | ○ |
| **B) In terms of** | |  | |  | |  | |  | |  |
| … | workload in self-employed settings | ○ | | ○ | | ○ | | ○ | | ○ |
| … | job satisfaction in self-employed settings | ○ | | ○ | | ○ | | ○ | | ○ |
| … | earning opportunities in self-employed settings | ○ | | ○ | | ○ | | ○ | | ○ |
| … | the cost-benefit ratio in self-employed settings | ○ | | ○ | | ○ | | ○ | | ○ |
| **Has today’s workshop changed your perception regarding the attractiveness of working as a general practitioner (GP)?** | | | | | | | | | | |
|  | | The attractiveness has … | | | | | | | | |
|  | | **-2**  clearly decreased | | **-1**  slightly decreased | | **0**  not changed | | **+1**  slightly increased | | **+2**  clearly increased |
| **A) In general** | | ○ | | ○ | | ○ | | ○ | | ○ |
| **B) In terms of** | |  | |  | |  | |  | |  |
| … | workload of general practitioners | ○ | | ○ | | ○ | | ○ | | ○ |
| … | job satisfaction of general practitioners | ○ | | ○ | | ○ | | ○ | | ○ |
| … | earning opportunities of general practitioners | ○ | | ○ | | ○ | | ○ | | ○ |
| … | the cost-benefit-ratio of general practitioners | ○ | | ○ | | ○ | | ○ | | ○ |
| Altogether, how satisfied are you with today’s workshop? | | | very unsatisfied | | rather unsatisfied | | rather satisfied | | very satisfied | |
|  |  |  | ○ | | ○ | | ○ | | ○ | |
| **In your own words …** | | | | | | | | | | |
| What were the most important insights for you from today’s course? | | | | | | | | | | |
| **Thank you for your kind support!** | | | | | | | | | | |
